# Supplementary material for: Asynchronous magnetic resonance elastography: Shear wave speed reconstruction using noise correlation of incoherent waves
Source: Magn Reson Med. 2022 Oct 27;89(3):990–1001. doi: 10.1002/mrm.29502 (PMC9792433; doi:10.1002/mrm.29502)
Supplement: Supplementary file 1 — DATA S1 MATLAB code used to generate the numerical simulations of Figure 1 [file MRM-89-990-s002.zip › k-Wave/helpfiles/writeAttributes.html]

writeAttributes :: Functions (k-Wave)


# writeAttributes

Write attributes to a k-Wave HDF5 file.

## Syntax

```
writeAttributes(filename)
writeAttributes(filename, file_description)
```

## Description

`writeAttributes` writes a set of additional attributes to the HDF5 file specified by the user. These attributes specify the file version, k-Wave version, type, and so on, and are required by the k-Wave C++ simulation code. If the `file_description` is not specified, a description is generated based on computer username and MATLAB version.

## Inputs

|  |  |
| --- | --- |
| `filename` | name of HDF5 file to write matrix to |
| `file_description` | custom file description |

## See Also

`h5writeatt`, `writeFlags`, `writeGrid`, `writeMatrix`
